# Household Poverty and Movement Behaviors in Preschool Children: A Longitudinal Study of Physical Activity, Screen Time, and Sleep

**DOI:** 10.3390/children13091132

**Published:** 2026-08-24

**Authors:** Priyanka Chaudhary, Jerraco L. Johnson, E. Kipling Webster, Peter T. Katzmarzyk, Amanda E. Staiano

**Affiliations:** 1 TSET Health Promotion Research Center, The University of Oklahoma Health Campus, Oklahoma City, OK 73104, USA; priyanka.chaudhary@pbrc.edu; 2 Pennington Biomedical Research Center, Baton Rouge, LA 70808, USA; peter.katzmarzyk@pbrc.edu; 3 Department of Kinesiology, Health Promotion, and Recreation, University of North Texas, Denton, TX 76203, USA; jerraco.johnson@unt.edu; 4 Department of Kinesiology, Recreation, and Sport Studies, The University of Tennessee, Knoxville, TN 37996, USA; kipwebster@utk.edu

**Keywords:** preschool children, household poverty, screen time, physical activity, sleep duration

## Abstract

**Highlights:**

**What are the main findings?**

- Children living at or below the federal poverty level had significantly higher screen time than children living above the poverty level at both baseline and 1-year follow-up.
- After adjusting for child characteristics, poverty status remained independently associated with approximately 2.8 additional hours of daily screen time at baseline. No statistically significant associations were detected for moderate-to-vigorous PA or nighttime sleep.

**What are the implications of the main findings?**

- Household poverty appears to be an important social determinant of excessive screen use during early childhood.
- Future studies should examine the contextual factors contributing to screen use among children from low-income households and evaluate strategies designed to reduce excessive exposure.

**Abstract:**

**Background:** Household poverty may shape physical activity (PA), screen time, and sleep during early childhood, but longitudinal evidence among preschool-aged children remains limited. This study examined longitudinal associations between household poverty status and these movement behaviors over 1 year. **Methods:** Data from 66 children aged 3–4.9 years were analyzed at baseline and 1-year follow-up. Parents reported sociodemographic characteristics and screen time, with poverty defined using household income and size. ActiGraph accelerometers (GT3X+) were worn for 7 days to estimate MVPA and nighttime sleep. Mixed-effects models examined associations between poverty status and each behavior, adjusting for child age, sex, race, and BMI percentile. Poverty status was assessed at each time point and treated as a time-varying exposure, and Time × Poverty interactions were included to test whether changes differed by poverty status. **Results:** At baseline, children living at/below the poverty level had higher screen time than children living above the poverty level (8.3 vs. 3.2 h/day, *p* < 0.05), and this descriptive difference remained at follow-up (7.0 vs. 3.1 h/day; *p* < 0.05). In adjusted linear mixed-effects models, children living at/below the poverty level had 2.8 additional hours/day of screen time at baseline (β = 2.84, 95% CI: 1.19–4.49, *p* = 0.001). The Time × Poverty interaction for screen time was not statistically significant (*p* = 0.64), suggesting no detectable difference in changes over time by poverty status. Poverty status was not significantly associated with MVPA or nighttime sleep, and their interactions with time were also not significant. **Conclusion:** Screen time was the only movement behavior independently associated with household poverty in this cohort. The non-significant findings for MVPA and nighttime sleep should be interpreted cautiously because of a small poverty subgroup and limited precision. Future studies should examine factors contributing to elevated screen use and evaluate strategies to reduce screen exposure among children from low-income households.

## 1. Introduction

Household poverty is a well-established social determinant of child health that shapes the environments in which physical activity (PA), screen time, and sleep develop during early childhood [1,2]. These behaviors often persist across the lifespan [3,4] and are important modifiable contributors to childhood obesity, which affects 12.7% of U.S. children aged 2–5 years and are associated with chronic disease and poor psychological outcomes [5,6,7]. Understanding how household poverty influences these behaviors is therefore important for developing interventions that support healthy routines among families.

PA is important for maintaining a healthy weight in young children [8,9], yet fewer than half of preschool-aged children meet the recommended 60 min of moderate-to-vigorous PA (MVPA) daily [10]. PA tends to decline as children grow older [11]. Children from lower socioeconomic status (SES) households may face structural barriers that limit their opportunities for PA, including limited access to safe outdoor spaces [12,13,14,15]. However, findings regarding socioeconomic differences in PA remain mixed, with some studies reporting no clear association and others identifying modest disparities [16,17]. This inconsistency highlights the need for prospective studies using objective measures to clarify whether socioeconomic differences in PA emerge or persist over time.

Excessive screen time is also concerning during early childhood because it has been linked to obesity, poor sleep, and adverse psychosocial outcomes [18,19]. The World Health Organization (WHO) recommends less than one hour of screen time per day for children aged 2 to 5 years [20], yet U.S. preschoolers average more than twice that amount [21]. Socioeconomic differences in screen use have also been reported, with children from lower-income or socioeconomically disadvantaged households consistently engaging in more daily screen time than their higher-income peers [22,23]. A recent national report showed that the screen time gap between children from lower- and higher-income families has reached nearly two hours per day [24]. Parents in lower-income households may also rely more heavily on screen media as a practical option for keeping children occupied and safe, especially where outdoor spaces are limited or unavailable, rather than out of a general preference for screen use [25,26]. Because PA and screen time may both be influenced by household socioeconomic circumstances, it is important to determine whether similar disparities are evident in sleep.

Insufficient sleep is another important behavior associated with obesity risk and poor health outcomes [27]. Inadequate or irregular sleep disrupts appetite regulation, increases caloric intake, and contributes to excess weight gain [28,29]. Evidence from a cohort study suggests that children from lower-income households may sleep roughly 30–40 min less per night than their higher-income peers, often due to environmental stress, inconsistent routines, or increased nighttime screen exposure [1,19]. Despite growing evidence linking SES to children’s sleep, only a few studies have examined this relationship using objective measures of sleep alongside PA and screen time behaviors.

Although prior studies have explored PA, screen time, and sleep separately, fewer have evaluated these behaviors together across household income groups. For instance, Anderson et al. (2008) found that approximately one in four U.S. children had both low PA and excessive screen time, while Lan et al. (2020) observed that each additional hour of screen use was linked to a 6 min reduction in sleep duration [30,31]. Some evidence also suggests that SES may influence multiple movement behaviors simultaneously [19,32]. However, most existing studies are cross-sectional limiting understanding of how socioeconomic disparities in these behaviors develop or change over time [3,32]. In addition, prior research has often relied on neighborhood-level socioeconomic indicators rather than household-level poverty classifications. Few studies have combined a household-level measure of poverty with repeated objective assessments of PA and sleep and parent-reported screen time among preschool-aged children. This distinction is important because household circumstances may directly influence children’s daily routines, access to resources, and opportunities to engage in healthy behaviors.

The present study addresses these gaps by examining household poverty as a time-varying exposure in relation to PA, screen time, and nighttime sleep across one year. This longitudinal household-level approach extends prior work by jointly evaluating repeated measures of three key movement behaviors in a U.S. preschool sample. Therefore, the purpose of this study was to examine the longitudinal associations between household poverty status and PA, screen time, and nighttime sleep among preschool-aged children over a 1-year period. We hypothesized that children living at or below the federal poverty level would have lower MVPA, higher screen time, and shorter nighttime sleep than children living above the poverty level and that these differences would remain evident across the follow-up period.

## 2. Materials and Methods

### 2.1. Study Design and Setting

This secondary analysis used data from the “Pause & Play” prospective cohort observational study, which involved preschool-aged children enrolled in ten early childhood education (ECE) centers. The parent study was designed to investigate the extent to which center-level policies and practices influenced children’s PA and screen time [33]. The present secondary analysis was conducted because previous Pause & Play published studies focused on ECE policy environments, PA, and a natural policy experiment involving center-level screen-time practices [33,34]. These studies did not examine household poverty as time-varying, family-level exposure or jointly evaluate repeated measures of PA, screen time, and nighttime sleep. The present study therefore extends the original work by providing a household-level longitudinal analysis of children’s movement behaviors.

Eligible centers were located East Baton Rouge Parish, Louisiana, and served children aged 3 to 5 years. Ten centers were randomly selected for participation, with stratification based on their licensing classification related to Child and Adult Care Food Program (CACFP) funding status. Participating centers included three church-based ECE centers, two Early Head Start or Head Start Centers (ECE centers that are federally funded to provide care to low-income children), and five centers that were corporate-sponsored, located in an elementary school, privately owned, or affiliated with a university [33]. Baseline data collection (April 2016 to January 2017) occurred before the implementation of a new state policy regarding screen time, and follow-up data were collected approximately one year later (May 2017 to May 2018) [34]. Of the ten ECE centers initially enrolled, one center participated only in baseline data collection and did not complete follow-up assessments. The study was approval by the Pennington Biomedical Research Center Institutional Review Board.

Families were recruited from participating ECE centers using at least two outreach methods, such as flyers, phone calls, emails, mailings, or in-person communication. To be eligible, children had to be between 3 and 4.9 years old, enrolled in full-time childcare (a minimum of 6 h per day), and expected to remain at the center one year later. Written parental consent was obtained, and study procedures were described to the preschoolers in age-appropriate terms, allowing them the option to decline participation. A total of 175 preschool-aged children were available for this secondary analysis. Of these, 109 were excluded, including 23 who had insufficient information to determine household poverty status, 16 who did not meet the accelerometer wear-time requirement of at least four valid days, and 70 who did not complete both the baseline and 1-year follow-up assessments. The final analytic sample included 66 children. A participant flow diagram is provided as Supplementary Figure S1.

### 2.2. Measures

Sociodemographic Information: Following consent, parents completed a demographic survey that collected details such as the child’s date of birth, sex, race/ethnicity, maternal education, household size, and household income. The child’s age was determined by calculating the time between the reported date of birth and the date of the assessment visit.

Anthropometric Measurements: Trained research staff measured each child’s height and weight. Height was measured to the nearest 0.1 cm using a portable stadiometer (Seca 213; seca GmbH & Co. KG, Hamburg, Germany), and weight was measured to the nearest 0.1 kg using a digital scale (Tanita 800S; Tanita Corporation, Tokyo, Japan). Each measurement was taken twice, and a third measurement was taken if the initial two differed by 0.5 units or more. Body mass index (BMI) percentiles and BMI z-scores were calculated based on the child’s age and sex using the U.S. 2022 CDC Growth Chart References [35].

Poverty Designations: Household poverty status was determined by comparing the parent-reported household income and household size to the U.S. Department of Health and Human Services Federal Poverty Guidelines at the time of data collection [36]. These guidelines account for the number of individuals living in the household to define income thresholds for poverty. Based on this comparison, families were categorized into two groups: (1) at/below the federal poverty level or (2) above the federal poverty level. Poverty status was reassessed at each assessment and was allowed to change between baseline and follow-up. Accordingly, poverty status was treated as a time-varying exposure in the longitudinal model.

Accelerometry: Children’s PA and sleep patterns were measured using an ActiGraph GT3X+ accelerometer (Ametris LLC, Pensacola, FL, USA). Trained research staff secured the device to the child’s right hip using an adjustable elastic band, and data were recorded in 15 s epochs [37]. Parents were instructed to ensure their child wore the accelerometer continuously for 24 h a day over a seven-day period, with an additional day provided for familiarization. The device was only to be removed for water-based activities, such as bathing. Accelerometer data were screened for implausible values and potential outliers using descriptive statistics and graphical inspection before analysis, and all observed values were plausible ranges for preschool-aged children.

Non-wear time was defined as any period of 30 min or more of consecutive zero counts per minute (cpm), in line with prior studies [38,39]. The 30 min criterion was selected to balance identification of true non-wear with the risk of incorrectly classifying periods of low movement as non-wear in young children, and such periods were excluded from the analysis. Data were processed using cut-points established by Pate et al. (2006) for 15 s epochs, categorizing activity levels as follows: sedentary (0–799 cpm), light PA (LPA: 800–1679 cpm), moderate PA (MPA: 1680–3367 cpm), and vigorous PA (VPA: ≥3368 cpm) [37]. MVPA was defined as ≥1680 cpm, and total PA (TPA) was calculated by summing LPA and MVPA. Although accelerometer data were collected in 15 s epochs, counts were expressed as counts per minute to align with published cut-points [37]. For inclusion in the analysis, children were required to have at least four valid days of accelerometer data, with a minimum of ten hours of wear time per day, consistent with previously published protocols [40,41].

Nighttime sleep duration was defined as the period between algorithm-determined bedtime and wake time. Children were included in the sleep-based analysis if they had at least three days with a minimum of 160 min of overnight sleep, which is the threshold required to identify a valid sleep phase based on established guidelines [42]. Only nighttime sleep was examined because the automated algorithm was validated for detecting nighttime sleep periods but not daytime naps in preschool-aged children. Because naps contribute to total sleep in this age group, their exclusion may underestimate 24 h sleep duration.

Screen time: Parents reported their child’s screen time using items adapted from the 2009–2010 National Health and Nutrition Examination Survey (NHANES), consistent with screen-time measures used in prior studies [43]. The primary item asked: “During the past 30 days, on average, how many hours did your child sit and watch television (TV) or videos outside of school?” Response options included: none, less than 1 h, 1, 2, 3, 4, 5, or more than 5 h. Responses were coded as follows: “none” as 0 h/day and “less than 1 h” as 0.5 h/day. This question format was repeated for four additional devices or platforms: computer/computer games, video games, smartphone use, and iPad/tablet use. No parent reported usage exceeding 5 h per day for any single device. Screen time was analyzed both by exposure to individual devices and as total daily screen time, calculated by summing reported hours across all devices. Because total screen time was derived by summing responses about specific devices rather than using a single overall screen-time item, concurrent use of multiple devices may have been counted more than once. Consequently, the resulting measure represents summed device-specific exposure and may overestimate mutually exclusive daily screen time.

Parents completed a sociodemographic survey that included questions on children’s screen time via hard copy, and all responses were entered by research staff into Research Electronic Data Capture (REDCap version 8.5.18; Vanderbilt University, Nashville, TN, USA), a secure, HIPAA-compliant data management platform used for research purposes [44,45].

### 2.3. Statistical Analysis

Descriptive statistics were calculated to summarize sociodemographic, anthropometric, and behavioral characteristics at baseline and 1-year follow-up, stratified by household poverty status (at/below vs. above the federal poverty level). Continuous variables were presented as means with standard deviations (SD) and categorical variables as frequencies and percentages. Between-group differences were evaluated using independent samples *t*-tests for continuous variables and chi-square tests or Fisher’s exact tests as appropriate for categorical variables.

Separate linear mixed-effects models with repeated measures were estimated for each behavioral outcome: (1) MVPA (minutes/day), (2) total screen time (hours/day), and (3) nighttime sleep (hours/night). Each model included fixed effects for time (baseline vs. follow-up), household poverty status (at/below vs. above the federal poverty level), child age, sex, race, and BMI percentile. Poverty status was assessed using parent-reported household income and household size and was therefore treated as a time-varying exposure. A child-level random intercept accounted for within-child correlation across repeated assessments.

A Time × Poverty interaction was included in each model to test whether changes from baseline to follow-up differed by poverty status. The interaction term directly evaluated whether the longitudinal pattern of each behavior varied between children living at/below versus above the poverty level. With baseline and above poverty specified as the reference categories, the poverty main effect represents the poverty-group difference at baseline, while the interaction represents the additional difference in change over time.

Because this was a secondary analysis of an existing cohort, no a priori sample-size calculation was conducted for the present research question. The analytic sample was determined by the number of participants with eligible data and sufficient information to classify household poverty status. The small number of children living at/below the poverty level limited statistical precision, particularly for the interaction effects and for detecting modest associations in MVPA and nighttime sleep. Therefore, non-significant findings are interpreted as an absence of statistically detectable associations rather than evidence of equivalence.

ECE-center clustering was not included in the primary models because the modest analytic sample and small number of participants within several centers did not support reliable estimation of an additional center-level random effect. The potential influence of unmodeled center-level clustering is addressed in the Limitations. Each model included only observations with complete data for the outcome, poverty status, and covariates. Missing values were not imputed.

Model results are presented as unstandardized beta coefficients (β) with standard errors (SE), 95% confidence intervals (CI), and corresponding *p*-values. Statistical significance was defined as *p* < 0.05 using two-sided tests. All analyses were conducted using IBM SPSS Statistical software version 29 (IBM Corp., Armonk, NY, USA).

## 3. Results

### 3.1. Participant Characteristics by Poverty Status

Descriptive characteristics of the sample by poverty status at baseline and 1-year follow-up are presented in Table 1. At baseline, the mean age was 3.0 ± 0.1 years, and at 1-year follow-up, the mean age was 4.0 ± 0.1 years. At baseline, 18.2% of children were living at/below the federal poverty level and 81.8% were living above the poverty level. At the 1-year follow-up, 13.6% children were living at/below the poverty level and 86.4% were living above the poverty level. Children living at/below the poverty level were predominantly African American (91.7%) and had lower maternal education than children living above the poverty level. The distribution of child sex differed significantly by poverty status at baseline (*p* < 0.05) but not at follow-up. At baseline, children at/below the poverty level also had a lower BMI percentile (42.2 ± 32.6 vs. 63.2 ± 27.3, *p* < 0.05), although this difference was not observed at follow-up. Household size and BMI z-scores were similar between groups at both baseline and follow-up.

PA levels did not differ significantly by poverty status, at either baseline or the 1-year follow-up (all *p* > 0.05), although descriptive differences were observed. Children living at/below the poverty level accumulated less MVPA at baseline (69.8 ± 40.3 vs. 93.0 ± 54.3 min/day) but more MVPA at follow-up (114.6 ± 15.0 vs. 67.6 ± 54.8 min/day) than children living above the poverty level. These descriptive differences were not statistically significantly different and should be interpreted cautiously given the small poverty subgroup. TPA and sedentary time were similar between groups at both time points.

In contrast, screen time was notably higher among children living at/below the poverty level at both time points. At baseline, children at/below the poverty level engaged in more than twice the total daily screen time of their peers (8.3 ± 6.0 vs. 3.2 ± 2.3 h/day; *p* < 0.05). These differences remained consistent at follow-up, with children at/below the poverty level continuing to average more than double the total screen time of those above the poverty level (7.0 ± 4.0 vs. 3.1 ± 2.2 h/day, *p* < 0.05). Parents of children at/below the poverty level reported greater television, smartphones, and iPads use. Because total screen time was calculated by summing responses across five device-specific questions, concurrent use of multiple devices may have contributed to higher total estimates.

Nighttime sleep duration was significantly shorter among children at/below the poverty level at baseline (9.9 ± 0.6 vs. 11.6 ± 1.9 h/night, *p* < 0.05) but did not differ significantly at the 1-year follow-up (11.3 ± 2.8 vs. 12.4 ± 1.6 h/night) (See Table 1).

### 3.2. Adjusted Associations Between Poverty Status and Movement Behaviors

Table 2 presents the fixed-effects estimates from the linear mixed-effects models examining associations between poverty status and children’s MVPA, total screen time, and nighttime sleep across the 1 year. Each model included a Time × Poverty interaction term for each outcome to assess whether changes from baseline to follow-up differed by poverty status. Models were adjusted for child age, sex, race, and BMI percentile. Reference categories were baseline, above the poverty level, girls, and White race.

Among children living above the poverty level, the reference group, changes from baseline to follow-up were not statistically significant for MVPA (β = 2.53, SE = 30.26, 95% CI: −56.8 to 61.8, *p* = 0.93), screen time (β = −1.84, SE = 1.10, 95% CI: −4.00 to 0.32, *p* = 0.09), or nighttime sleep (β = −0.65, SE = 0.84, 95% CI: −2.28 to 0.99, *p* = 0.44).

The Time × Poverty interactions were also not statistically significant for all three outcomes, including MVPA (β = 52.00, SE = 42.63, 95% CI: −31.6 to 135.6, *p* = 0.22), screen time (β = −0.36, SE = 0.75, 95% CI: −1.83 to 1.12, *p* = 0.64), or nighttime sleep (β = −0.49, SE = 0.86, 95% CI: −2.18 to 1.20, *p* = 0.57). These findings indicate no statistically detectable evidence that changes in the three behaviors differed by poverty status over the 1-year period. Given the small poverty subgroup and wide confidence intervals, the interaction estimates should be interpreted cautiously.

As the models included a Time × Poverty interaction, the poverty main effect represents the difference between poverty groups at baseline. Children living at/below the poverty level had 2.8 additional hours of daily screen time at baseline compared with children living above the poverty level (β = 2.84, SE = 0.84, 95% CI: 1.19 to 4.49, *p* = 0.001). However, poverty status was not significantly associated with MVPA (β = −24.38, SE = 33.63, 95% CI: −90.3 to 41.5, *p* = 0.47) or nighttime sleep (β = −0.72, SE = 0.75, 95% CI: −2.19 to 0.76, *p* = 0.34) at baseline.

Race was also associated with screen time. African American children had 1.66 additional hours of daily screen time compared with White children (β = 1.66, SE = 0.69, 95% CI: 0.31 to 3.10, *p* = 0.01). Children in the “other” race category had 4.82 additional hours of daily screen time compared with White children (β = 4.82, SE = 1.60, *p* = 0.02). Race was not significantly associations with MVPA or nighttime sleep.

Child age was positively associated with longer nighttime sleep (β = 1.81, SE = 0.86, 95% CI: 0.13 to 3.49, *p* = 0.04). No other covariates, including sex and BMI percentiles, were significantly associated with MVPA, total screen time, or nighttime sleep. The relatively wide confidence intervals for the MVPA and nighttime sleep estimates indicated limited precision and reduced ability to detect modest associations for these outcomes.

## 4. Discussion

The present prospective study found that household poverty was independently associated with greater screen time among preschool-aged children. After adjustment for child age, sex, race, and BMI percentile, children living at/below the federal poverty level accumulated approximately 2.8 additional hours of screen time per day at baseline than children living above the poverty level. The non-significant interaction indicated that this disparity remained relatively stable over the 1-year follow-up. In contrast, poverty status was not significantly associated with MVPA or nighttime sleep, and neither outcome showed a significant interaction with time. These findings extend prior cross-sectional and neighborhood-level socioeconomic research by demonstrating a persistent household-level difference in screen use during a developmentally sensitive period. In contrast, poverty status was not significantly associated with MVPA or nighttime sleep, and neither outcome had significant interaction with time. Because the small poverty subgroup and limited precision for these outcomes, the non-significant findings should be interpreted cautiously and not as evidence that the groups were equivalent.

While children in both groups exceeded the WHO screen time use recommendation of <1 h/day for preschool-age children, the screen time observed among children living at/below the federal poverty level, approximately 7 to 8 h/day at baseline and remained approximately 7 h/day at follow-up, was significantly higher than typical estimates reported for U.S. preschoolers [20,46]. Other U.S.-based studies have also reported average preschool screen time ranging from 95 to 122 min/day, suggesting that excessive screen exposure remains a widespread challenge or concern for all children [47,48]. Most similar to the geographic location of the current sample, Webster and colleagues (2019) found that preschoolers in Louisiana accumulated an average of 5.1 h/day of total screen exposure across multiple screen devices [49]. More recently, Ouyang et al. (2023) found that preschoolers’ screen time nearly doubled during the COVID-19 pandemic in an international sample [50]. It is also important to note that, in the present study, screen time was assessed separately for multiple device types and then summed, which may have contributed to higher total estimates. This approach may be influenced by recall bias, particularly if children used multiple devices concurrently, potentially leading to overestimation of total screen time. Nevertheless, the same measurement approach was applied across poverty groups. The greater screen time observed among children living at/below the poverty level may reflect differences in household routines, available recreational resources, caregiver demands, or other circumstances associated with economic hardship. These potential mechanisms were not directly measured and should be investigated in future studies.

Beyond total screen exposure, our descriptive results also found some differences in how screen time was accumulated across device types. Children from households below the poverty level reported greater use of different types of screen devices, such as TV, smartphones, and iPads, whereas children living above the poverty level appeared to rely somewhat more on television viewing. Nonetheless, these findings are broadly consistent with national data indicating that lower-income households increasingly rely on portable media devices as the primary source of entertainment and engagement for children [32,47]. Such device use shifts are critical because mobile and touchscreen media are more immersive and less co-viewed, potentially displacing social play and outdoor activity. Prior studies have shown that children from lower-SES families are more likely to use screens for passive entertainment or unsupervised play, while those from higher-SES backgrounds more often use educational or co-viewed content [51,52]. However, the present study did not assess the content, context, timing, or degree of caregiver involvement in screen use. Consequently, conclusions cannot be drawn regarding whether devices use displaced active play, involved educational content, or occurred with caregiver co-viewing. Future studies should examine these dimensions alongside total exposure.

High screen use among children living in poverty may reflect structural and environmental constraints rather than solely parental choice. Screen media may sometimes serve as an accessible way for caregivers to manage competing demands, particularly when safe play spaces, affordable recreational opportunities, or predictable work schedules are limited [1,8]. Further, findings from qualitative studies had similarly described screen media as a practical tool for managing household responsibilities and stress. Because these factors were not assessed in the present study, they should be considered possible explanations rather than established mechanisms. Future intervention studies should evaluate whether practical, family-centered strategies can reduce screen exposure while accounting for the constraints experienced by low-income households.

Importantly, despite the observed difference in screen time, we did not detect statistically significant differences in MVPA between children living at/below and above the federal poverty level. Children in both groups averaged at least 60 min of MVPA per day at both time points. However, the descriptive pattern differed across assessments: children living at/below the poverty level had lower MVPA at baseline but higher MVPA at follow-up than children living above the poverty level. Because these descriptive differences were not statistically significant, changed direction over time, and were based on a small poverty subgroup, they may reflect sampling variability or changes in subgroup composition rather than a consistent socioeconomic pattern. Larger longitudinal studies are needed to determine whether poverty is associated with preschool children’s PA trajectories One possible, although untested, explanation is the shared ECE environment. All children attended ECE centers, and prior research suggests that structured center-based routines may reduce socioeconomic differences in preschool children’s MVPA in some settings [53,54]. However, the present study did not directly evaluate ECE policy implementation or center-level differences in PA opportunities, this interpretation should be viewed as a possible explanation rather than a tested mechanism.

Similarly, most children met the recommended nighttime sleep duration ranges [55]. Although adjusted models did not detect statistically significant differences in nighttime sleep between poverty groups, these findings should again be interpreted cautiously, given the small number of children in the poverty subgroup and the exclusion of daytime naps from the sleep estimates. Structured ECE routines may have contributed to the comparable nighttime sleep duration observed across poverty groups. However, because ECE characteristics and center-level differences were not examined, this explanation remains speculative. Nonetheless, prior research indicates that other dimensions of sleep, including sleep quality, timing, and regularity, may be affected by household conditions, caregiver stress, and bedtime screen exposure [1,56]. These factors were not captured by the nighttime sleep-duration measure used in this study. Future studies with larger and more diverse samples should include daytime naps, sleep timing, sleep regularity, and sleep quality to more comprehensively examine socioeconomic differences in preschool children’s sleep.

From a public health perspective, screen time was the only movement behavior independently associated with household poverty in this cohort. This finding identifies screen use as a potential focus for future research and intervention development. Family-centered strategies may need to consider structural barriers to alternative forms of play and provide practical, affordable options for reducing screen exposure [57,58]. However, the effectiveness of such strategies was not evaluated in the present study and should be examined in future intervention research.

### Strengths and Limitations

There are several strengths to note. The study’s prospective cohort design with repeated measures over one year allows for the examination of within-child change over time, offering stronger inference than cross-sectional analyses. Children’s movement behaviors were captured objectively with ActiGraph GT3X+ accelerometers using 15 s epochs, yielding granular estimates of MVPA and nighttime sleep while minimizing recall bias. Household poverty status was operationalized using parent-reported income and household size aligned with federal poverty guidelines, enabling policy-relevant classification of poverty at the family level rather than reliance on neighborhood proxies. Screen exposure was measured by device type (TV, computer, video games, smartphone, iPads), allowing identification of disparities concentrated in mobile/touchscreen media that can inform targeted intervention components. The sampling frame included ECE centers spanning Head Start/Early Head Start, church-based, corporate, school-based, and university-affiliated settings, enhancing ecological validity across diverse care environments.

Several limitations warrant consideration. First, although no statistical differences were observed between children included and excluded from the analytic sample, participant attrition may still have introduced selection bias. Second, the relatively small number of children living at/below the federal poverty level limited the precision of the MVPA and sleep estimates and reduced the ability to detect modest differences or Time × Poverty interactions. Third, participants were recruited from multiple ECE centers, however, center-level clustering was not included in the statistical models because of the modest analytical sample and limited number of children within individual centers. In addition, none of the Time × Poverty interactions were statistically significant. However, the small poverty subgroup limited the power and precision of these interaction tests. Finally, residual confounding remains possible because factors such as household routines, parental work schedules, neighborhood environments, and screen use practices were not measured.

## 5. Conclusions

Children living at/below the federal poverty level had greater screen time than children living above the poverty level, and the magnitude of this difference did not change significantly over the one-year follow-up. Screen time was the only movement behavior independently associated with household poverty in this cohort. No statistically significant associations were detected for MVPA or nighttime sleep, although these findings should be interpreted cautiously because of the small poverty subgroup and limited precision. Future studies should investigate the contextual factors contributing to elevated screen use and evaluate whether family- and community-based interventions can reduce screen exposure among children living in low-income households.

## Figures and Tables

**Table 1:** Baseline and 1-Year Follow-up Characteristics of Children by Poverty Level (N = 66).

Variables	Baseline		1-Year Follow-Up	
	At/Below Poverty (N = 12; 18.2%)	Above Poverty (N = 54; 81.8%)	At/Below Poverty (N = 9; 13.6%)	Above Poverty (N = 57; 86.4%)
Age (years), mean (SD)	3.0 (0.0)	3.0 (0.1)	4.0 (0.0)	3.9 (0.1)
Sex (%) Boy Girl	50.0 * 50.0 *	48.1 51.9	55.6 44.4	47.4 52.6
Race (%) White African American Other	0.0 91.7 8.3	83.3 13.0 3.7	0.0 88.9 11.1	82.5 15.8 1.7
Maternal Education (%) Less than High School/GED Some Colleges or Higher	75.0 * 25.0 *	1.9 89.1	66.7 33.3	7.0 93.0
Household size, mean (SD)	4.0 (1.2)	3.7 (0.8)	3.7 (0.6)	3.7 (0.9)
BMI z-score, mean (SD)	−0.2 (1.2)	0.4 (0.9)	−0.2 (1.2)	0.4 (1.2)
BMI percentile, mean (SD)	42.2 (32.6) *	63.2 (27.3)	48.6 (35.1)	60.4 (28.3)
Physical Activity (min/day) mean (SD) LPA MVPA TPA Sedentary	226.4 (99.6) 69.8 (40.3) 296.3 (135.9) 312.1 (95.4)	195.7 (97.7) 93.0 (54.3) 288.8 (143.9) 307.5 (136.8)	228.5 (35.0) 114.6 (15.0) 343.1 (50.0) 276.3 (15.3)	175.0 (104.8) 67.6 (54.8) 242.1 (153.5) 259.8 (140.6)
Screen Time (h/day), mean (SD) TV Computer Video Games Smartphones iPad Total screen time	2.7 (1.3) * 1.3 (1.8) 1.2 (1.6) 1.3 (1.4) * 1.7 (1.8) * 8.3 (6.0) *	1.5 (0.9) 0.3 (0.5) 0.2 (0.3) 0.4 (0.4) 0.7 (0.8) 3.2 (2.3)	3.2 (1.2) * 0.4 (1.0) 0.8 (1.2) 1.4 (1.5) * 1.0 (1.2) 7.0 (4.0) *	1.4 (0.9) 0.3 (0.4) 0.2 (0.4) 0.5 (0.5) 0.7 (0.9) 3.1 (2.2)
Nighttime Sleep (h/night) mean, (SD)	9.9 (0.67) *	11.6 (1.9)	11.3 (2.8)	12.4 (1.6)

Values are presented as mean (SD) for continuous variables and percentages for categorical variables. * Indicated statistically significant differences between poverty groups at *p* < 0.05. LPA = light-intensity physical activity; MVPA = moderate-to-vigorous physical activity; TPA = total physical activity. SD = standard deviation; h/day = hours per day; h/night = hours per night; GED = General Education Development.

**Table 2:** Adjusted Fixed Effects Estimates for Children’s MVPA, Screen Time, and Sleep, including Time × Poverty Interaction.

Variables	MVPA (β [SE]) [95% CI]	*p*-Value	Screen Time (β [SE]) [95% CI]	*p*-Value	Sleep (β [SE]) [95%CI]	*p*-Value
Time (Follow-up)	2.53 (30.26) [−56.8, 61.8]	0.93	−1.84 (1.10) [−4.00, 0.32]	0.09	−0.65 (0.84) [−2.28, 0.99]	0.44
Poverty (At/Below Poverty)	−24.38 (33.63) [−90.3, 41.5]	0.47	2.84 (0.84) [1.19, 4.49]	0.001 *	−0.72 (0.75) [−2.19, 0.76]	0.34
Time × Poverty	52.0 (42.63) [−31.6, 135.6]	0.22	−0.36 (0.75) [−1.83, 1.12]	0.64	−0.49 (0.86) [−2.18, 1.20]	0.57
Sex (Boys)	4.96 (15.31) [−25.1, 35.0]	0.75	−0.54 (0.67) [−1.84, 0.77]	0.42	0.31 (0.46) [−0.60, 1.22]	0.50
Race (African American)	14.74 (25.65) [−35.5, 65.0]	0.57	1.66 (0.69) [0.31, 3.10]	0.01 *	−0.97 (0.59) [−2.13, 0.19]	0.10
Race (Other)	−13.59 (42.89) [−97.7, 70.5]	0.75	4.82 (1.60) [1.68, 7.96]	0.02 *	1.10 (1.02) [−0.91, 3.10]	0.28
Age (years)	−30.04 (31.16) [−91.1, 31.0]	0.34	1.71 (1.13) [−0.50, 3.92]	0.13	1.81 (0.86) [0.13, 3.49]	0.04 *
BMI percentile	−0.002 (0.30) [−0.58, 0.58]	0.99	−0.015 (0.010) [−0.03, 0.004]	0.11	−0.008 (0.007) [−0.02, 0.006]	0.27

Estimates are unstandardized regression coefficients (β) with standard errors in parentheses and 95% confidence intervals in brackets. Models include fixed effects for time, poverty status, Time × Poverty, sex, race, age, and BMI percentile, with a child level random intercept. Reference categories are baseline, above the federal poverty level, girls, and White race. Significant results (*p* < 0.05) are * indicates between group differences.

## Data Availability

The data that support the findings of this study are not publicly available due to ethical and privacy restrictions but may be available from the corresponding author upon reasonable request and with appropriate institutional approvals.

## Supplementary Materials

The following supporting information can be downloaded at: https://www.mdpi.com/article/10.3390/children13091132/s1, Figure S1: Flow diagram of participant selection for the present secondary analysis.
