# Supplementary material for: Household Poverty and Movement Behaviors in Preschool Children: A Longitudinal Study of Physical Activity, Screen Time, and Sleep
Source: Children (Basel). 2026 Aug 24;13(9):1132. doi: 10.3390/children13091132 (PMC13605833; doi:10.3390/children13091132)

Supplemental Figure S1. Flow diagram of participant selection for the present secondary analysis

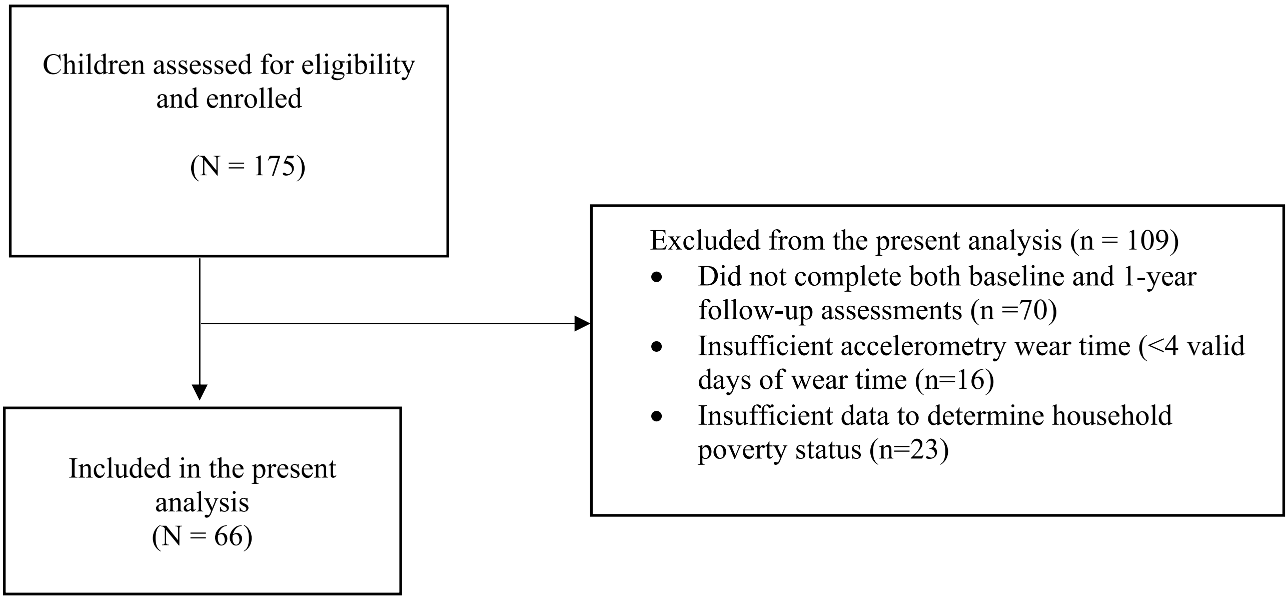

Supplement: Supplementary file 1 [file children-13-01132-s001.zip › children-4445726-supplementary.pdf]
